# From Duke to King's: Michael Malim wins the 2010 Retrovirology prize

**DOI:** 10.1186/1742-4690-7-103

**Published:** 2010-12-01

**Authors:** Ariberto Fassati

**Affiliations:** 1 The Wohl Virion Centre and Medical Research Council Centre for Medical & Molecular Virology, Division of Infection and Immunity, University College London, UK

## Abstract

Michael H. Malim wins the Retrovirology prize.

## 

Retrovirology awards an annual Prize to recognize outstanding achievements in the field by mid-career scientists, older than 45 but younger than 60 [1]. The prize is supported through a donation from the Ming K. Jeang Foundation, an educational foundation based in Houston, Texas, USA. This year is the 6^th ^edition of the prize, alternating yearly between recognizing a non-HIV retrovirologist and an HIV retrovirologist. Previous Awardees are Steve Goff [2], Jo Sodroski [3], Karen Beemon [4], Ben Berkhout [5] and Thierry Heidman [6] and although we maintain strict confidentiality about nominees who did not win, many of them were also outstanding scientists who may well receive the prize in the future, testament to how much the Retrovirology Prize is truly valued by our community.

For 2010 the Editors have awarded the Retrovirology Prize to Michael H. Malim (Figure 1). Mike is Professor and Head of the Department of Infectious Diseases at King's College London, UK. He is a Fellow of the Academy of Medical Sciences, an EMBO member since 2005 and a Fellow of the Royal Society since 2007. Mike graduated in Biochemistry from Bristol University (UK) and obtained his DPhil from Oxford University working on Ty retrotransposition. He then moved to the US at Duke University, where he worked with Bryan Cullen as a postdoctoral fellow and then, in 1992, set up his own lab at the University of Pennsylvania. In 2001, he returned to the UK at King's College London. Amongst his very many scientific achievements I would like to highlight two. In the late'80s and early '90s Mike, together with Bryan Cullen, showed that the HIV-1 accessory protein Rev was required for nuclear export of unspliced viral RNA and that it acted by binding to a highly structured RNA stretch called the Rev-response element [7,8]. This represented a tremendous leap forward in understanding HIV-1 biology. It also had important implications outside the HIV field because at the time not much was known about mRNA export mechanisms and unspliced mRNA was not supposed to be exported from the nucleus [9]. Thanks in part to Mike's contributions we then understood that HIV-1, via Rev, evolved to recruit CRM1, a nuclear export receptor, to bypass the normal cellular mRNA export process and have its unspliced mRNA transported to the cytoplasm for translation and packaging. A remarkable exploitation of the cellular machinery!

**Figure 1:**
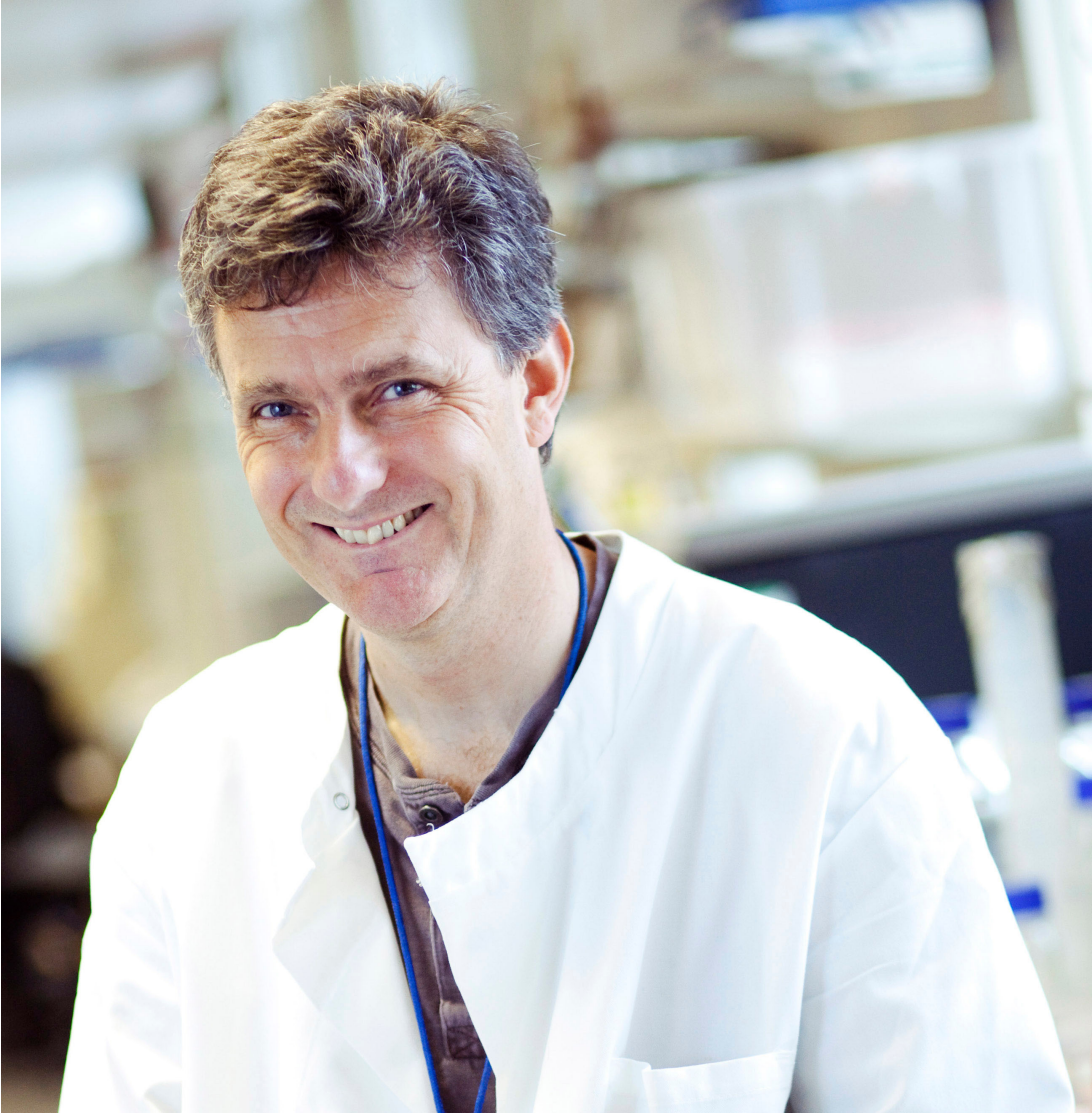
**Prof. Michael Malim**.

In the mid '90s he turned his attention to another HIV accessory protein called "viral infectivity factor" or Vif. Several groups, including his own, had shown that Vif-deficient HIV-1 could not replicate in certain cell types, including peripheral blood mononuclear cells (non-permissive cells) but could replicate in others (permissive cells). Despite substantial effort by several groups, the function of Vif remained elusive for many years. In 1998, Mike's group using hybrids of permissive and non-permissive cells showed that the non-permissive phenotype was dominant and hence its activity very likely depended on a host cell factor [10]. The quest for this factor continued in Mike's lab for several years, despite the skepticism of colleagues. His efforts were eventually vindicated in 2002 when Anne Sheehy in his group reported that the factor being overcome by Vif was APOBEC3G, a single stranded DNA cytidine deaminase [11]. APOBEG3G was shown to hypermutate the viral genome during reverse transcription and also to impair reverse transcription itself and irreversibly damage the provirus [12]. We then understood that Vif induced degradation of APOBEC3G, preventing its incorporation into nascent viral particles. The discovery of the antiviral activity of APOBEC changed our understanding of innate immunity to retroviruses and retroelements in general.

As it is customary, I have asked Mike to share with us some thoughts on his own work and on general issues of interest to the community.

AF: When did you become interested in Science?

MHM: I suppose I always enjoyed science subjects at high school. I particularly remember an inspiring chemistry teacher, who also used to teach us to play bridge. I studied biochemistry as an undergraduate, and my enthusiasm for research was ignited by a pretty unsuccessful research project in fungal genetics. Nevertheless, this persuaded me to pursue a PhD.

AF: Why did you decide to work on HIV-1?

MHM: It was during my post-graduate research in the mid-1980s that HIV-1 was first isolated and characterised. I recall being amazed by all the novel genes the virus contained, and the unpredictable functions they seemed to have. I decided to try and do post-doctoral research in the area, and have continued to work in that area to this day - it has been an incredible experience!

AF: What do you consider your most important scientific contributions?

MHM: Hmm... I think that two areas to which my (or more accurately, our) research has contributed significantly are in understanding the functions of the viral Rev and Vif proteins. The work with Bryan on Rev and RNA transport was an amazing time: in fact, when we started in 1987, nuclear export was not really considered to be a regulated process. The research on Vif leading to the discovery of APOBEC3G, and its assignment as a DNA mutator, was equally exciting: I still find it pretty astonishing that one single protein can have such a profound inhibitory effect on HIV infection.

AF: Is there any contribution of yours you feel has been neglected?

MHM: No, not really, I feel the HIV literature gets picked over pretty intensely, and so it is quite difficult for an instructive paper to fly under the radar screen.

AF: Can you tell us about current research in your laboratory?

MHM: We remain pretty focused on HIV-host interactions. We continue to work on APOBEC3 proteins, aspects of their anti-viral mechanism, their impact on natural HIV infection, and potential effects on host cell function. A second area of interest, which has brought us back to RNA nuclear export, is how the history of viral mRNA can influence the process of HIV particle assembly - a project that was stimulated by trying to understand post-integration blocks to HIV replication in non-human cells. And, more recently, we have begun to explore the mechanism(s) that cells use to register HIV infection as well as the interplay between type 1 interferon and HIV replication.

AF: Cross-disciplinary science is often praised but seldom funded: do you have any advice for scientists who want to bridge different fields?

MHM: I think this will become increasingly important in biological and medical research; the advent of new technologies and approaches drive fields forwards and often depends on folks trained in disparate areas - maths and physics to name just two that often crop up in conversation. To promote meaningful interactions, I think there are (at least) two key elements: good communication and pump-prime funding. My own biases are that the former often proceed more productively through personal contact, and the latter needs institutions and funding agencies to think creatively about how they can provide modest initial support for "blue skies" ideas.

AF: You have worked on both sides of the Atlantic: what are the lessons that you learnt in the US and what the ones that you learnt in the UK?

MHM: It can be pretty dangerous to make sweeping generalisations about the idiosyncrasies of different nations! However, I think the differences in science culture between the two countries are quite subtle these days. I would venture that scientists in the US are less constrained by the concerns of failure and have a bit more of a "can do" attitude. For example, I think that US labs are often happier to confront very ambitious projects: though part of this is probably also due to the longer amount of time that PhD students are allowed to get their degrees. On the other hand, my own experience has been that the UK seems a slightly easier place to collaborate... though I acknowledge this might be attributable to shifts in my own perspectives as time goes by!

AF: How important is mentorship to you?

MHM: I think that having people to turn to for advice on important issues is essential, and I find there are attributes to admire and aspire to in all sorts of people. However, my belief is that mentoring works best on an informal basis and that you need different folks for help in different areas (you wouldn't ask your butcher about how to fix your second serve). Indeed, I think seeking and providing counsel are central to building and supporting a community - such as science and education. I am not a great fan of formal mentorship schemes where you have to rock up for your annual mentorship meeting with someone you barely cross paths with, and who has little clue about what you do (even if this does tick an absurd box in the institutional procedure manual). So, I am always happy to provide advice to junior/senior colleagues on almost anything, but hope that they balance my views with those of others.

AF: What do you most value in your colleagues?

*MHM: Among both institutional and fellow scientists, I suppose I value a commitment to the broader of community - we are not working in isolation, and co-operation and collegiality, even when in competition, are vital. For scientists, I certainly admire those with creativity and dedication, blended with a healthy sense of irreverence*.

AF: The Retrovirology prize is awarded to "mid career" scientists: what do you think are the specific challenges facing this group of colleagues?

MHM: These are different for all of us! I am a strong believer that we are only as good as our last paper, and that we therefore need to continue to strive for impact, innovation and quality. I think this is what gets us out of bed in the morning, and will enable us to contribute in the face of all the talent that is out there. For me, personally, a major challenge is balancing the demands of running a mid-size research group with burgeoning administrative and institutional roles and responsibilities. The latter can be very rewarding, especially as one sees new programmes and initiatives develop, and is certainly of great importance to the future success of respective institutions, but it can be consuming at times: I think compartmentalisation has to be the key.

AF: You are Editor of several journals, including one open-access. What is your experience as an Editor and what do you think are the most significant changes that open-access publication brought about?

MHM: As you know yourself, editing can be very rewarding, especially when you have the opportunity to shape the future of a journal. I was one of the initial Editors of PLoS Pathogens (2005), and I am chuffed with the success of this journal. It has certainly filled an important niche that did not exist previously in pathogen-related research. Working with the crop of volunteer Editors we have had for HIV/AIDS has been a privilege. Beyond the content of this particular journal, I am a great supporter of open-access publishing. The idea that anyone, anywhere can read about, and benefit from, the science that is being sponsored by public funds seems fundamental to the scientific ethos. It is gratifying that so many researchers have embraced this notion and send so much of their better work to open-access journals.

AF: Students in the near future, at least in the UK, will leave university with a substantial debt burden. Those who embark in a career in science will struggle for many years on low paid jobs and insecurity about future prospects. Can you give them good reasons why they should go into science?

MHM: Correct, these are certainly testing times. But, funding has dipped before, and such matters are cyclical so I remain optimistic that they will recover. As for embarking upon research as a career, it has so much to recommend it. It is never dull: it is hard to match the challenge and buzz of discovery and learning, the constant renewal of lab personnel with new perspectives and ideas is always invigorating, and teaching/training can be incredibly rewarding. While the hours can be long, they are also very flexible, and I think this makes it a career with excellent compatibility with family life. I have also been fortunate to make many lifetime friends from all over the world, and conferences always provide good venues to put one's efforts into broader perspective, catch up, and, perhaps, even raise a glass or two!

AF: Who would you like to thank?

MHM: Several sets of people: the labs I have trained in, the institutions and funders who have supported me, various colleagues, and the wonderful trainees, fellows and staff who have worked in my lab. And, of course, my family for their continuing tolerance.
